# First Case of *Raoultella planticola* Urinary Tract Infection Reported in Western Romania

**DOI:** 10.3390/medicina59030506

**Published:** 2023-03-04

**Authors:** Alin Gabriel Mihu, Monica Maria Susan, Carmen Nicoleta Strauti, Maria Daniela Mot, Horia Dan Muresanu, Cornel Balta, Alexandru Nesiu

**Affiliations:** 1 Bioclinica Medical Analysis Laboratory, 310300 Arad, Romania; 2 “Aurel Ardelean” Institute of Life Sciences, Vasile Goldis Western University of Arad, 310414 Arad, Romania; 3 Department of Internal Medicine, Victor Babes University of Timisoara, 300041 Timisoara, Romania; 4 Preventive Medicine Research Centre, Victor Babes University of Timisoara, 300226 Timisoara, Romania; 5 Intensive Care Unit 2, Arad County Emergency Clinical Hospital, 310037 Arad, Romania; 6 Faculty of Medicine, Vasile Goldis Western University of Arad, 310414 Arad, Romania; 7 Department of Urology, Arad County Emergency Clinical Hospital, 310037 Arad, Romania

**Keywords:** urinary tract infection, UTI, rare, *Raoultella planticola*

## Abstract

*Raoultella planticola* is a Gram-negative bacterium rarely involved in urinary tract infections. The patient was an 80-year-old woman with several associated diseases who presented to the hospital with fever and dysuria. *Raoultella planticola* was identified to be the causative agent of the urinary tract infection. Antibacterial treatment led to a full recovery within 7 days. This report highlights the presence of a rare pathogen as a causative agent in the case of a urinary tract infection and also the importance of using multiple methods in order to identify bacteria and to establish the diagnosis.

## 1. Introduction

Urinary tract infections (UTIs) are responsible for significant morbidity and increased health care costs, representing the most common infectious presentation in community medical practice, after respiratory tract infections [1]. Studies suggest that worldwide, each year, more than 150 million people are affected by UTIs, affecting both males and females of all age groups throughout their life span. Mostly, these infections require antibacterial therapy in addition to treatment with prebiotics, probiotics and urinary antiseptics [1,2].

The most common isolated pathogens in the case of community-acquired uncomplicated UTIs, both in males and females, are *Escherichia coli*, being the most common, followed by *Enterobacteriaceae* (*Klebsiella*, *Proteus*, *Enterobacter*), *Enterococci*, *Streptococci*, *Staphylococci* and *Pseudomonas* spp. [3,4].

Currently, *Raoultella* spp. was recognized as an important emerging uropathogen and should be considered in cases of infection [5]. *Raoultella planticola* is a Gram-negative, non-motile, aerobic, encapsulated bacterium belonging to the *Enterobacteriaceae* family [6]. This bacterium is usually found in aquatic environments, plants and soil. The cases of infection in humans caused by this organism were described as bacteremia, pneumonia, cholangitis, abscesses and UTIs [7]. Most of the patients infected by this pathogen present a degree of immunosuppression and malignancies, and have experiences of chemotherapy, organ transplants, prolonged ICU hospitalization or recent trauma [8].

In order to exert its pathogenicity in the human body, this bacterium has developed several mechanisms, such as the ability to adhere to the tissues for which it has developed tropism, form biofilms to protect itself and transform amino acid histidine into histamine, a mechanism that leads to the appearance of local and general clinical manifestations [9].

Due to these reasons, we present the first case report in Romania of an 80-year-old female, diagnosed with a symptomatic, lower UTI caused by *Raoultella planticola*.

## 2. Materials and Methods

### 2.1. Patient

An 80-year-old female with type II diabetes and stage II hypertension presented to the Department of Urology from Arad County Hospital with a history of a 3-day fever (38.7 °C) and symptoms of dysuria, polyuria and pressure in the lower abdomen (hypogastric region). A complete blood count (CBC) and urine analysis as well as urine culture, blood glucose, hemoglobin A1c (HbA1c) and inflammatory markers in the form of C reactive protein (CRP) and the erythrocyte sedimentation rate (ESR) were analyzed. An abdominal ultrasound was also performed.

### 2.2. Specimen Collection

Blood samples were collected via cephalic vein puncture on hematology vacutainers with K3 EDTA and on biochemistry vacutainers with a clot activator and separating gel in order to perform hematological and biochemical analyses.

The patient was instructed to self-collect the mid-stream of urine using the clean-catch technique in a sterile container.

### 2.3. Laboratory Assays

CBC was determined using flow cytometry, cytochemistry and spectrophotometry on Advia 2120i (Siemens Healthcare Diagnostics, Erlangen, Germany).

Blood glucose (using stectrophotometry) and CRP (using imunotubidimetry) were determined on Cobas c501 (Roche Diagnostics International Ltd., Rotkreuz, Switzerland). HbA1c was measured using high-performance liquid chromatography Tosoh HLC-723GX analyser (Tosoh Corporation, Tokyo, Japan). 

ESR was determined using the automated analyzer Roller 20PN (Alifax S.p.A., Polverara, Italy).

Urine biochemistry was determined using the automated dipstick method on CLINITEK^®^ Novus Analyzer (Siemens Healthcare Diagnostics, Erlangen, Germany).

Urine sediment was determined using an automated urine microscopy analyzer, iQ200 Series Analyzer (Beckman Coulter, Brea, CA, USA).

### 2.4. Microbiology Techniques and Assays 

The urine was plated using a 10 μL calibrated inoculating loop with a cross-streak pattern to conventional chromID CPS Elite chromogenic agar (bioMérieux Inc., Craponne, France) within one hour from the collection. The culture was incubated for 24 h in ambient air, at 37 °C. 

The chromeID media were read and interpreted in accordance with the criteria in the standard operating procedure for urine cultures in the clinical microbiology laboratory. A growth of one or two distinct organisms above 50,000 CFU/mL was considered clinically significant. In our case, one organism (>100,000 CFU/mL) was further isolated into pure colonies [10].

A bacterial suspension was adjusted to 0.50 McFarlands in a solution of 0.45% sodium chloride. Bacterial isolates were further inoculated into the appropriate VITEK identification and antibiogram strips using VITEK^®^ 2 Compact (BioMérieux, Inc., Hazelwood, MO, USA). Analysis was performed using the identification (card type: GN) card and an antibiotic card (card type: AST-N204) for Gram-negative bacteria [11]. The data obtained were analyzed using VITEK 2 software version 9 in accordance with the manufacturer’s instructions.

Matrix-assisted laser desorption/ionization time-of-flight (MALDI-TOF) microbial identification was performed on VITEK MS (BioMérieux, Inc., Hazelwood, MO, USA) in accordance with the manufacturer’s instructions and internal laboratory standards. 

### 2.5. Ethical Approval

Ethical approval of the clinical protocol for this study was approved by the Ethics Committee of Arad County Emergency Clinical Hospital (no. 90/4.01.2023), and the informed consent was signed by the patient.

## 3. Results

Laboratory tests were significant for the high level of white blood cells (WBC) count within the CBC of 17.100/mm^3^ (normal: 4.050–11.840/mm^3^), with an 88% neutrophil (normal: 42–77%) count which presented toxic granules and 3% unsegmented neutrophils. CRP was elevated to 3.2 (normal: <0.5 mg/dL) and the ESR was slightly elevated to 33 mm/h (normal: <30 mm/h). Blood glucose and HbA1c were slightly raised at 125 mg/dL (normal: 82–115 mg/dL) and 6.8% (normal: 4–6%).

Urine biochemistry revealed a yellow urine with cloudy clarity with a specificity gravity test of 1.019 (normal: 1.005–1.030), pH of 5.5 (normal: 4.6–8), trace for occult blood (normal: negative), moderate leukocyte esterase (normal: negative) and positive nitrites (normal: negative). 

**Figure 1:**
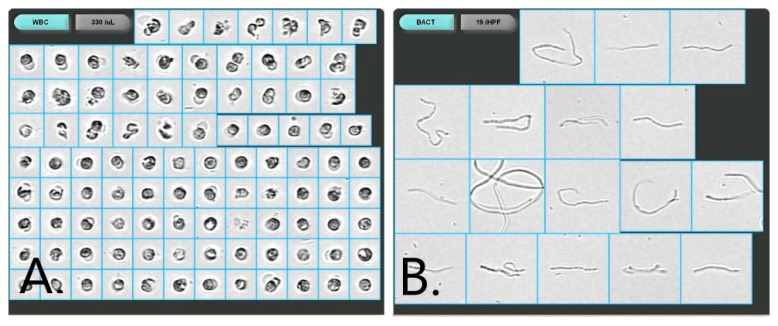
Urine sediment examination (**A**) Urine microscopy presenting WBCs and (**B**) long filamentous rods (automated microscopy, iQ200, ×400).

Automated urine microscopy revealed an abnormal value of white blood cells (WBC) of 330/μL (normal: <28/μL) (Figure 1A) and 19 bacteria/HPF (normal: <1/HPF) (Figure 1B). 

**Figure 2:**
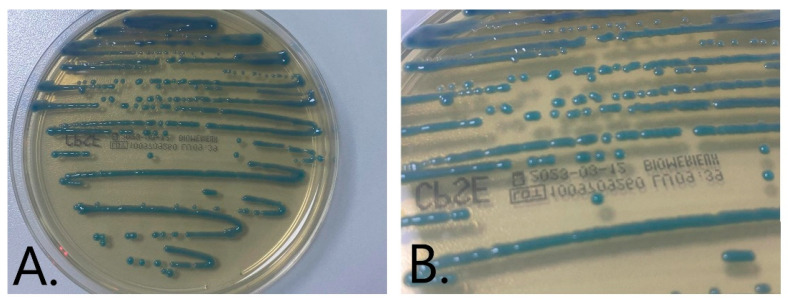
(**A**,**B**) Isolated green-to-blue colonies on chromID CPS Elite (bioMérieux) medium from the primary culture.

Urine culture was performed on chromID CPS Elite (bioMérieux) media where green-to-blue colonies belonging presumptive to the KESC group (*Klebsiella*, *Enterobacter*, *Serratia*, *Citrobacter*) developed (Figure 2A,B).

**Figure 3:**
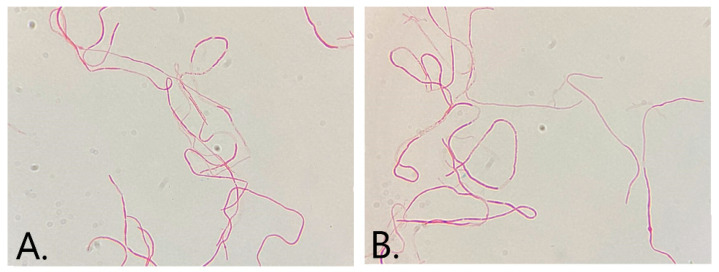
(**A**,**B**) Gram stain of the pure isolated bacterial colonies presenting long filamentous Gram-negative rods (×1000).

A Gram stain performed from the pure culture isolated on chromID media revealed long, filamentous Gram-negative bacilli and further proceeded to identify this bacterium (Figure 3A,B).

The Gram-negative rod was identified to be *Raoultella planticola* using VITEK^®^ 2 system and then confirmed via MALDI–TOF (matrix-assisted laser desorption ionization–time-of-flight). Further antibacterial susceptibility was commenced on VITEK^®^ 2 system and revealed sensibility to Amoxicillin/Clavulanic Acid, Piperacillin/Tazobactam, Cefotaxime, Ceftazidime, Cefepim, Ertapemen, Imipenem, Meropenem, Amikacin, Gentamicin, Ciproprofloxacin, Nitrofurantoin, Trimethoprime + sulfamethoxazole and resistance to Ampicillin (Table 1). 

Patient was treated with Tagremin 400 mg (Trimetoprime + sulfamethoxazole) 2 × 2 daily for 7 days and the evolution was conditionally and progressively improved after the first 2 days of treatment.

One week after ending the treatment, we repeated the CBC and inflammatory markers. The WBC count normalized at 8700/mm^3^ (normal: 4050–11,840/mm^3^), with 60% neutrophils as well as CRP at 0.3 mg/dL (normal: <0.5 mg/dL). ESR moderately improved to 27 mm/h (normal: <30 mm/h). The patient was asymptomatic. The abdominal ultrasound revealed no significant abnormal findings.

## 4. Discussion

An 80-year-old female with multiple medical-associated pathologies presented to the hospital with severe cystitis caused by *Raoultella planticola*. This bacterium was rarely associated with infection, most often being reported as a colonizer, especially in newborns [12]. Due to the fact that data regarding *Raoultella planticola* as a pathogen in humans are limited, the mechanism of pathogenesis is currently unclear. Certain conditions such as immunocompromised states, dialysis-dependencies, malignancy, diabetes mellitus and certain medication such as proton pump inhibitors and chemotherapy increase the chances of infection [9,13].

*Raoultella planticola* is a bacterium mostly found in environments with high prevalence in water and soil, first described in 1985 under the names *Klebsiella planticola* and *Klebsiella trevisanii* [14].Originally, *Raoultella planticola* was classified as a member of the genus *Klebsiella*, but since 2001, based on 16S rRNA and rpoB gene sequencing, this bacterium was reclassified as *Raoultella* spp. [15]. *Raoultella planticola* can convert histidine into histamine and can cause symptoms of scombroid poisoning when inadequately prepared seafood is consumed in large quantities [9].The first case where *Raoultella* spp. was incriminated as a pathogenic bacterium was reported in 1984 in a patient with sepsis [15]. In 2013, Olson et al. [12] reported the first case of UTI caused by this organism in an 89-year-old male patient with a past history of biventricular heart failure, chronic kidney disease, coronary artery disease, obesity, hypertension, anemia, atrial fibrillation and a penicillin allergy. 

Fager et al. [5] reported thirty-two serious cases of human infections with *Raoultella* spp. between 1984 and 2018, and just six cases were associated with UTIs. Other studies [16,17] reported dysuria and fever as symptomatology in the case of UTI-associated *Raoultella planticola* infection, similar to our clinical case report. 

To date, it is difficult to differentiate between *Klebsiella* spp. and *Raoultella* spp. via light microscopy analysis, due to their similar morphological and tinctorial characteristics [18]. Our microscopical investigation reported long filamentous Gram-negative rods for *Raoultella planticola*, which is a non-regular characteristic and not shown in another study. However, long filamentous morphotypes of *Enterobacteriaceae* were reported in the case of intensive bacterial cell multiplication [19].

Different biochemical tests are used to differentiate between *Raoultella* spp. and *Klebsiella* spp., such as tests focusing on ornithine decarboxylase activity, D-melezitose utilization and histamine [20,21]. In our study, we used VITEK^®^ 2 compact automated system microbial identification (ID), a system with 47 biochemical tests designed to identify Gram-negative rods within approximately 10 h [18,22]. However, this automatic system can confuse the *Raoultella* spp. With *Klebsiella* spp. [23]. Due to this reason, we continued with a second diagnosis using the MALDI-TOF equipment. MALDI-TOF has a better sensitivity of 97.4% compared to 93.3% on VITEK^®^ 2 compact automated system, where *Raoultella* spp. was firstly identified [18].Another study conducted in 2019 which used different assays in order to identify 30 strains of *Raoultella planticola* found MALDI-TOF equipment (with a sensitivity of 98.88% and specificity of 57.89%) to be superior over the VITEK^®^ 2 (with a sensitivity of 95.12% and specificity of 14.93%) [24].

The bacterium identified as a pathogen in our patient was resistant to Ampicillin. The resistance of *Raoultella planticola* to this antibiotic was previously reported due to over-expression of the encoded class-A β-lactamase [16,23,25]. This resistance is well known in the case of *Klebsiella spp*. and former *Klebsiella* members as *Raoultella planticola* (formerly known as *Klebsiella planticola*) [26].

*Raoultella planticola* is generally sensitive to antibacterial medication belonging to the aminoglycoside, carbapenem, cephalosporin and fluoroquinolone categories [16,27]. Treatment with a one-week course of Tagremin 400 mg (2 × 2 daily) led to the full recovery of the patient. Due to the fact that the control urine culture after the antimicrobial treatment is recommended only in females with recurrent UTIs one to two weeks after the end of the antimicrobial treatment [28], and considering the patient did not mention anything about recurrent UTIs in the initial anamnesis, we chose to follow the European Association of Urology Guideline and not perform a second urine culture in an asymptomatic patient [29]. The inflammatory markers were repeated one week after the end of the treatment and were in the normal range.

## 5. Conclusions

We described a rare case of UTI with *Raoultella planticola* in Western Romania, a Gram-negative bacterium usually misdiagnosed as *Klebsiella* spp. due to its morphology on chromogenic culture medium. We also observed that this bacterial species has gained resistance to ampicillin.

We also highlighted the importance of using biochemical tests on automated analyzers, such as the VITEK^®^ 2system in order to accurately identify an isolated pure colony obtained from the patient’s urine.

In conclusion, *Raoultella planticola* highlights a real clinical interest because it shows pathogenicity and develops the ability to become resistant to antibiotics.

## Figures and Tables

**Table 1:** Susceptibility results obtained in vitro on VITEK^®^ 2 system for *Raoultella planticola* (isolated pure culture obtained from urine, US pump and isotonic solution).

Antibacterial	MIC * (mg/L)	Interpretation
Ampicillin	16	R
Amoxicillin/Clavulanic Acid	≤2	S
Piperacillin/Tazobactam	≤4	S
Cefotaxime	≤1	S
Ceftazidime	≤1	S
Cefepim	≤1	S
Ertapemen	≤0.5	S
Imipenem	1	S
Meropenem	≤0.25	S
Amikacin	≤2	S
Gentamicin	≤1	S
Ciproprofloxacin	≤0.25	S
Nitrofurantoin	≤16	S
Trimetoprime + sulfamethoxazole	≤20	S

* MIC, minimum inhibitory concentration.

## Data Availability

All data are included in the manuscript.
